# Data on evolution of intrinsically disordered regions of the human kinome and contribution of FAK1 IDRs to cytoskeletal remodeling

**DOI:** 10.1016/j.dib.2016.11.099

**Published:** 2016-12-08

**Authors:** Jaymin J. Kathiriya, Ravi Ramesh Pathak, Alexandr Bezginov, Bin Xue, Vladimir N. Uversky, Elisabeth R.M. Tillier, Vrushank Davé

**Affiliations:** a Morsani College of Medicine, Department of Pathology and Cell Biology, University of South Florida, Tampa, FL 33612, USA; b Department of Cancer Biology and Evolution, H. Lee Moffitt Cancer Center and Research Institute, Tampa, FL 33612, USA; c Department of Medical Biophysics, University of Toronto, Toronto, Ontario, Canada; d Department of Cell Biology, Microbiology and Molecular Biology, University of South Florida, Tampa, FL 33620, USA; e Department of Molecular Medicine, Morsani College of Medicine, University of South Florida, Tampa, FL 33612, USA; f USF Health Byrd Alzheimer׳s Research Institute, University of South Florida, Tampa, FL 33612, USA; g Laboratory of Structural Dynamics, Stability and Folding of Proteins, Institute of Cytology, Russian Academy of Sciences, St. Petersburg, Russian Federation

**Keywords:** Focal adhesion Kinase-1, Protein kinases, Evolution of intrinsically disordered regions, Cytoskeletal remodeling

## Abstract

We present data on the evolution of intrinsically disordered regions (IDRs) taking into account the entire human protein kinome. The evolutionary data of the IDRs with respect to the kinase domains (KDs) and kinases as a whole protein (WP) are reported. Further, we have reported its post translational modifications of FAK1 IDRs and their contribution to the cytoskeletal remodeling. We also report the data to build a protein-protein interaction (PPI) network of primary and secondary FAK1-interacting hybrid proteins. Detailed analysis of the data and its effect on FAK1-related functions have been described in “Structural pliability adjacent to the kinase domain highlights contribution of FAK1 IDRs to cytoskeletal remodeling” (Kathiriya et. al., 2016) [1].

**Specifications Table**

**Table:** 

Subject area	*Biology*
More specific subject area	*Bioinformatics, Computational and Systems Biology, Evolutionary Biology*
Type of data	*Tables and Figures*
How data was acquired	*Computational Analysis*
Data format	*Raw and Filtered*
Experimental factors	*None*
Experimental features	*Intrinsically disordered regions of the human protein kinome have been computationally analyzed.*
Data source location	*N/A*
Data accessibility	*With this article*

**Value of the data**

• This data includes detailed information on evolution of each of the IDRs in the entire human protein kinome, which can be leveraged to study either individual IDRs within a kinase or a group of IDRs within a kinase family.
• Protein-protein interaction (PPI) data reported here can be utilized to further explore the contribution of IDRs in facilitating kinase functions.
• Our data on the utility of IDRs by FAK1 to relay its cytoskeleton-related signaling can lead to future experiments to determine the role of IDRs in the entire process of cytoskeletal remodeling.
• KD-adjacent IDRs, which are under higher evolutionary pressure, can be further analyzed to identify functionally important regions within these IDRs for future therapeutic targeting of kinases.

## 1 Data

Data reported here are related to the article entitled “Structural Pliability Adjacent to the Kinase Domain Highlights Contribution of FAK1 IDRs to Cytoskeletal Remodeling” [1]. Six figures and nine tables are presented in this article. The figures illustrate the function of IDRs in FAK1 and its effects on cytoskeletal remodeling. The tables provide raw data utilized to build PPI networks. Evolutionary scores of IDRs, kinase domains, and whole kinases are also reported in tables. One single Microsoft Excel file is provided with one table on each of the nine sheets (Fig. 6).

## 2 Experimental design, materials and methods

### 2.1 Disorderliness in the kinome

**Fig. 1:**
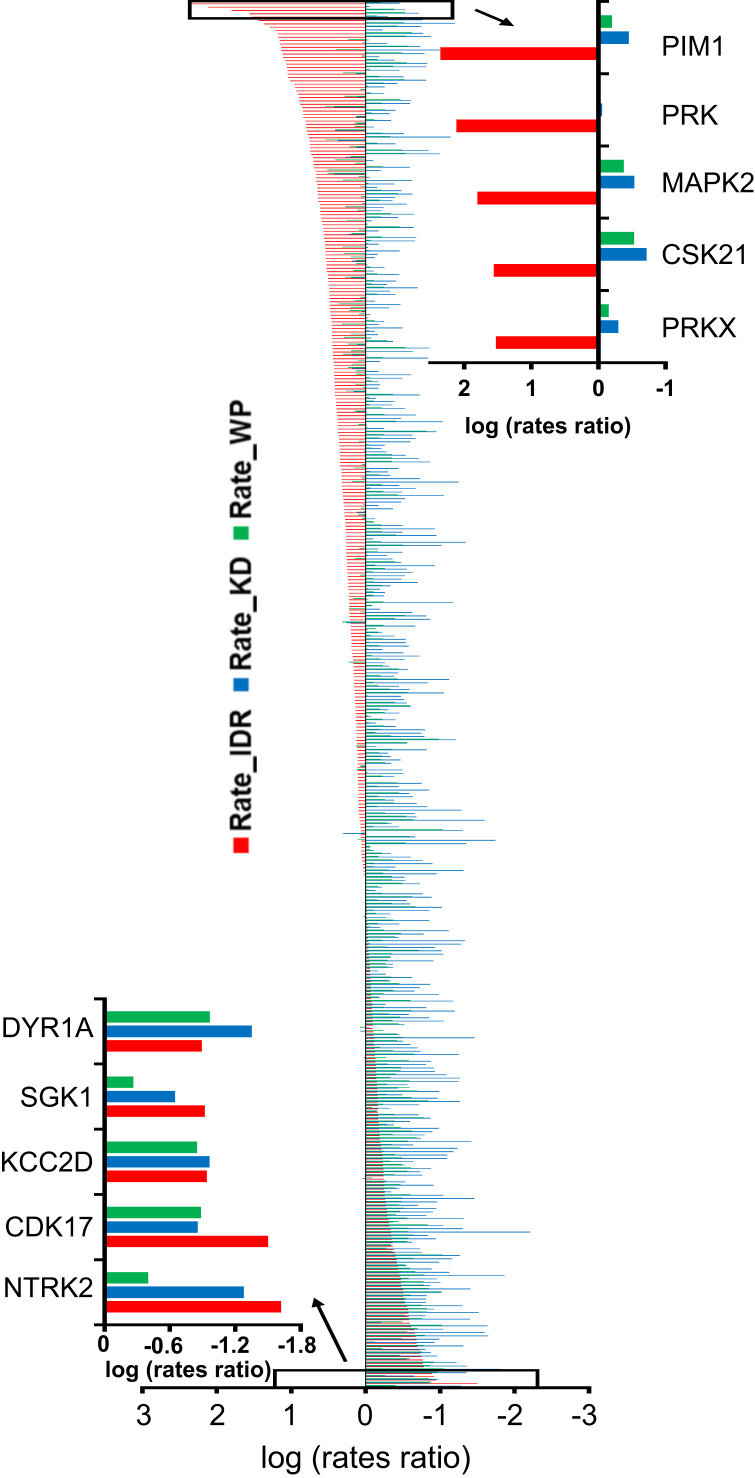
*Evolution of IDRs, KDs, and whole kinases (WP):* The rates of evolution are depicted in the bar graph for each kinase: WP (green), its KD (blue), and average of all the IDRs (red) for each kinase. A total of 414 kinases are depicted after they were sorted on the basis of the lowest to highest evolutionary rates of their IDRs. For brevity, we have also included the bottom inset depicting five kinases with lowest evolutionary rates of their IDRs. Likewise, the top inset depicts five kinases with highest evolutionary rates of their IDRs. The boxes in the main image depict the location of the top and bottom insets.

**Fig. 2:**
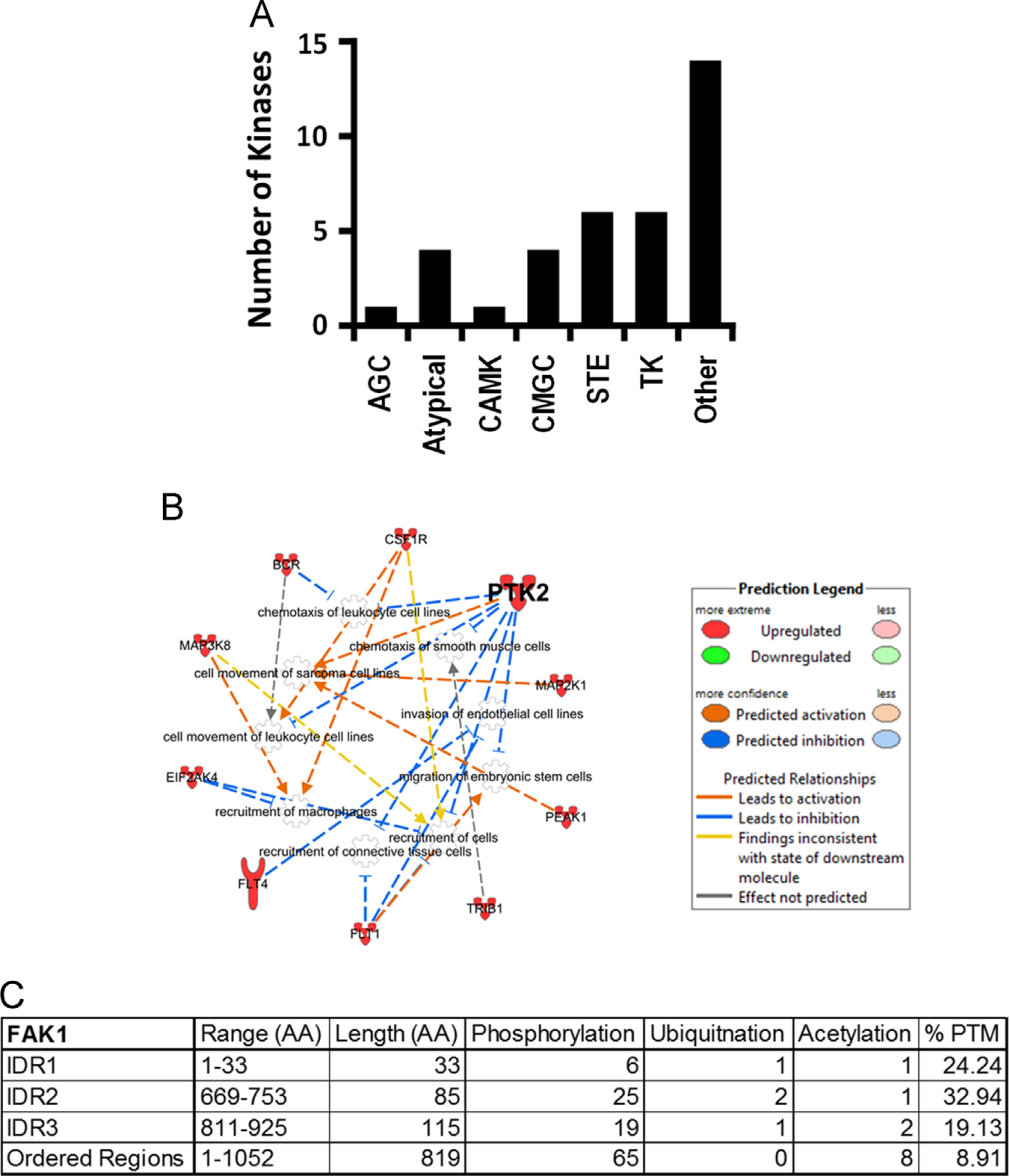
Identification of 36 Kinases with IDRs extending into the KDs and cellular migration as a significantly enriched molecular function. (A) 36 kinases were identified which had their IDRs extending into the KDs. Here, their distribution across seven different kinase groups is depicted. Raw data are provided in Supplementary Tables 4 and 5. (B) We identified cellular migration as a significantly enriched molecular function via the set of 36 kinases when compared to rest of the kinome (Fig. 4C in Ref. [1]). Network of proteins involved in cellular migration is depicted with predicted inter-relationships. Red color-filled molecules are part of 36 kinases. (C) Post translation modifications (PTM) were predicted in IDR_1_ (flanking the KD), IDR_2_ (2^nd^ closest IDR to the KD), IDR_3_ (N-terminal region, which is farthest away from the KD), and rest of the ordered regions of FAK1. These PTMs include phosphorylation, acetylation, and ubiquitination. % PTM was calculated as described in methods.

We predicted intrinsic disorder in the human kinome using Pondr-FIT software [2] (Figs. 1 and 2A; Supplementary Table 1). PONDR-FIT is an artificial neural network-aided meta-predictor of disordered residues. PONDR-FIT combines output of 7 different individual disorder predictors to increase confidence of disorder prediction by an average of 11% as compared to individual disorder predictors [2]. PONDR-FIT utilizes the following amino acid characteristics to predict disorder residues: Amino composition, amino acid sequence complexity, amino acid position specific scoring matrices, hydrophobicity and net charge of amino acid sequence, and pairwise interaction energy between amino acids of a given protein We considered the residues with disorder scores of ≥0.5 to have structure breaking propensities, or as we call it intrinsically disordered residues, as previously described [3]. A long disordered region with a stretch of at least 25 such amino acids constituted an IDR in our analysis. Previously reported disorder prediction of 504 kinases was used to calculate the fraction of total disordered amino acids in each kinase [4]. An amino acid labeled with disorder score of 0.5 or greater was considered as contributing to protein disorder. The total number of disordered amino acids was divided by the total number of amino acids present in a given kinase to calculate % DO (percent disorder) in the kinome. KINOMErender [5], a visualization tool for overlapping annotations on a phylogenic tree of protein kinases annotated the kinome dendrogram with % DO for each of the 504 kinases. We have excluded proteins without confirmed kinase domains in UniProt database. Kinome dendrogram illustration was reproduced, courtesy of Cell Signaling Technology, Inc. (www.cellsignal.com). Intrinsic disorder prediction of FAK1 and its orthologs was performed using Pondr-FIT [2], IUPred-L [6], IUPred-S [6], VSL2 [7], VSL3 [3], VLXT [8], Espritz [9], PrDOS [10].

### 2.2 Evolutionary analysis of kinases, KDs, and IDRs

The relative rates of evolution for the proteins and their domains (Fig. 1, Fig. 2; Supplementary Tables 1–3) were calculated as described by Kathiriya et al. [1].

### 2.3 PTM analysis of IDRs

We predicted phosphorylation sites using disPhos [11] and netphos [12], acetylation sites using PAIL [13], and ubiquitylation sites using UbPred [14]. These PTM predictors, in addition to predicting novel PTM sites, also predict and report the PTM sites that are already experimentally validated. We have cross-validated the data to ensure that all the PTM data reported for FAK1 in UniProt are included in our PTM data generated using these softwares. We calculated the abundance of PTM sites and normalized it to the total length of ordered or disordered regions as per the following equation (Fig. 2C):

$$\%\;\mathrm{PTM}\;=\;\frac{\mathrm{Phosphorylation}\;\mathrm{Sites}\;+\;\mathrm{Ubiquitination}\;\mathrm{Sites}\;+\;\mathrm{Acetylation}\;\mathrm{Sites}}{\mathrm{Lengt}h\;\mathrm{of}\;\mathrm{the}\;\mathrm{region}\;\left(\mathrm{AA}\;\mathrm{residues}\right)}x100$$

### 2.4 Network and functional analysis

**Fig. 3:**
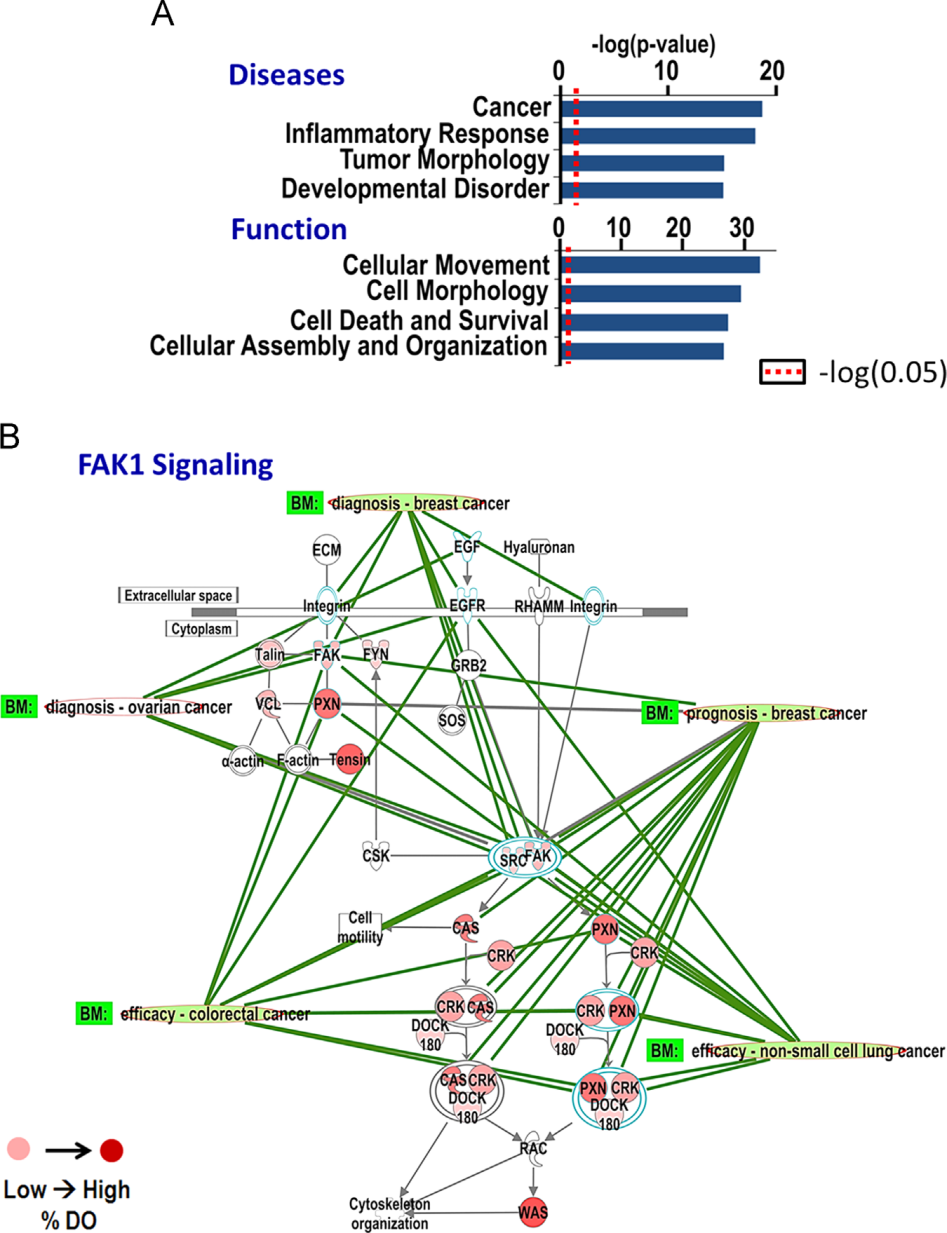
FAK1 signaling is mediated by hybrid proteins. (A) Functional analysis of the 98 proteins revealed that FAK1 relays its functions of modulating cellular movement, morphology and organization through its primary interactome, which enriches important diseases such as cancer. Analysis was performed using IPA. Red dotted line represents the threshold for significance (p<0.05). (B) FAK1 signaling pathway was enriched by the FAK1 primary interactome. Overlaying percent disorderliness of hybrid proteins revealed that cytoskeleton organization pathways are modulated by FAK1 interacting protein partners, most being intrinsically disordered. Intensity of red color denotes percent disorder in a protein in this pathway. The signaling pathway was further analyzed to identify proteins that serve as clinical markers for various cancer types. Intrinsically disordered hybrid proteins were identified as important biomarkers for a number of cancers.

Core analysis of 36 kinases was performed using Ingenuity Pathway Analysis (IPA) as described by Kathiriya et al. [15] (Fig. 3). Network of cellular migration as a significantly enriched function of the 36 kinases was identified. Disease and functional enrichment was performed as described by Kathiriya et al. [4].

### 2.5 Derivation of PPIs

**Fig. 4:**
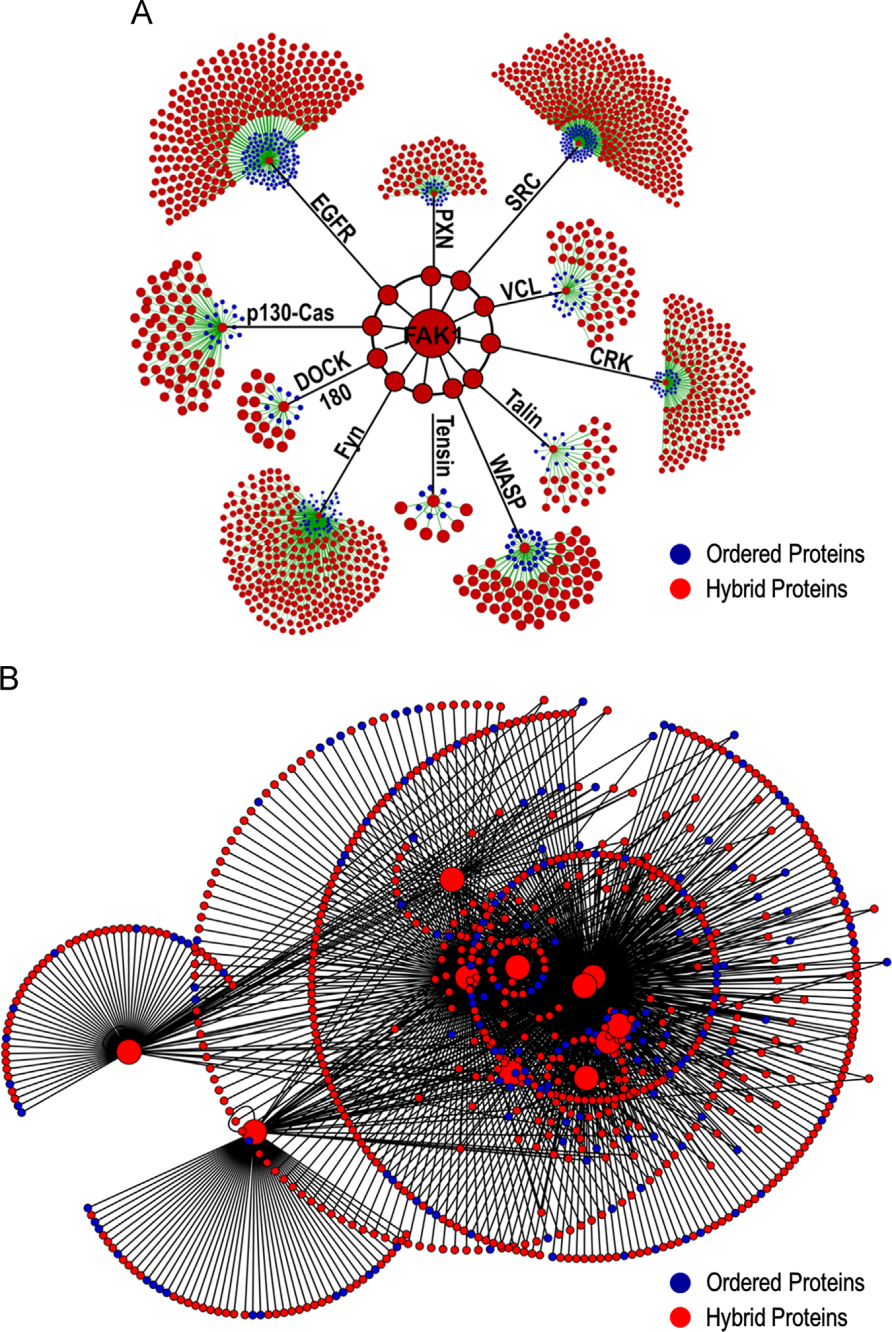
Intrinsic disorder provides structural pliancy in cytoskeletal remodeling via FAK1 and its secondary interactome. (A) Primary interactomes derived from 11 hybrid FAK1 interacting cytoskeletal proteins formed the FAK1 secondary interactome. Our analysis identified that 77% of FAK1 secondary interactome is comprised of hybrid proteins. Blue circles represent structured proteins and red circles represent hybrid proteins. (B) Overlay of the 11 interactomes onto one another revealed a high degree of crosstalk. 30% of the proteins interacted with at least 2 of the 11 cytoskeletal hybrid proteins while 27 proteins interacted with at least 5 of the 11 cytoskeletal hybrid proteins, indicating that these 11 cytoskeletal hybrid proteins are major hub proteins governing the cytoskeletal remodeling network.

**Fig. 5:**
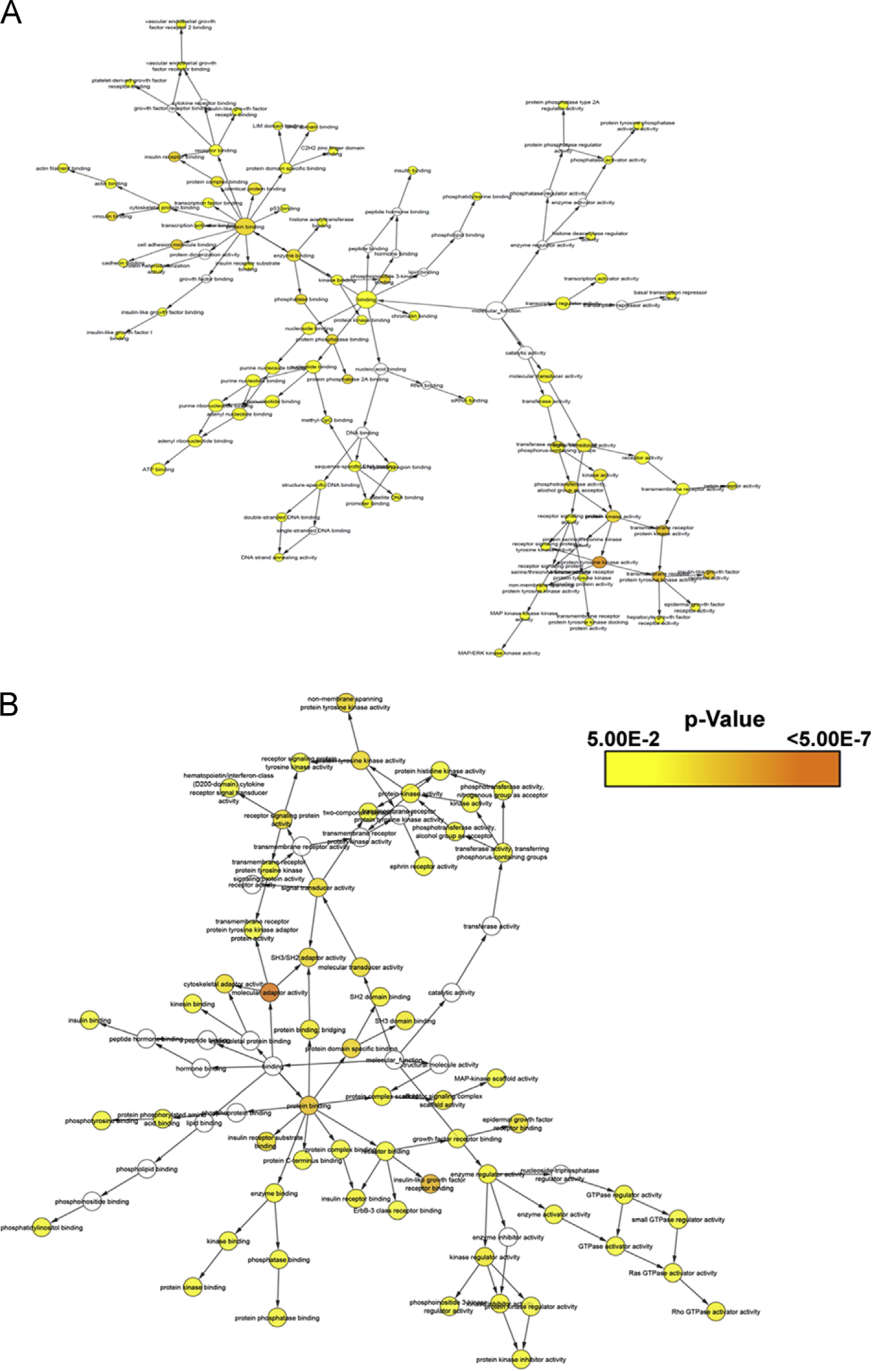
Molecular processes of the mapped interactome of FAK1. Molecular functions of (A) the proteins interacting with ordered regions and (B) the proteins interacting with IDRs were identified using BiNGO and visualized by Cytoscape.

**Fig. 6:**
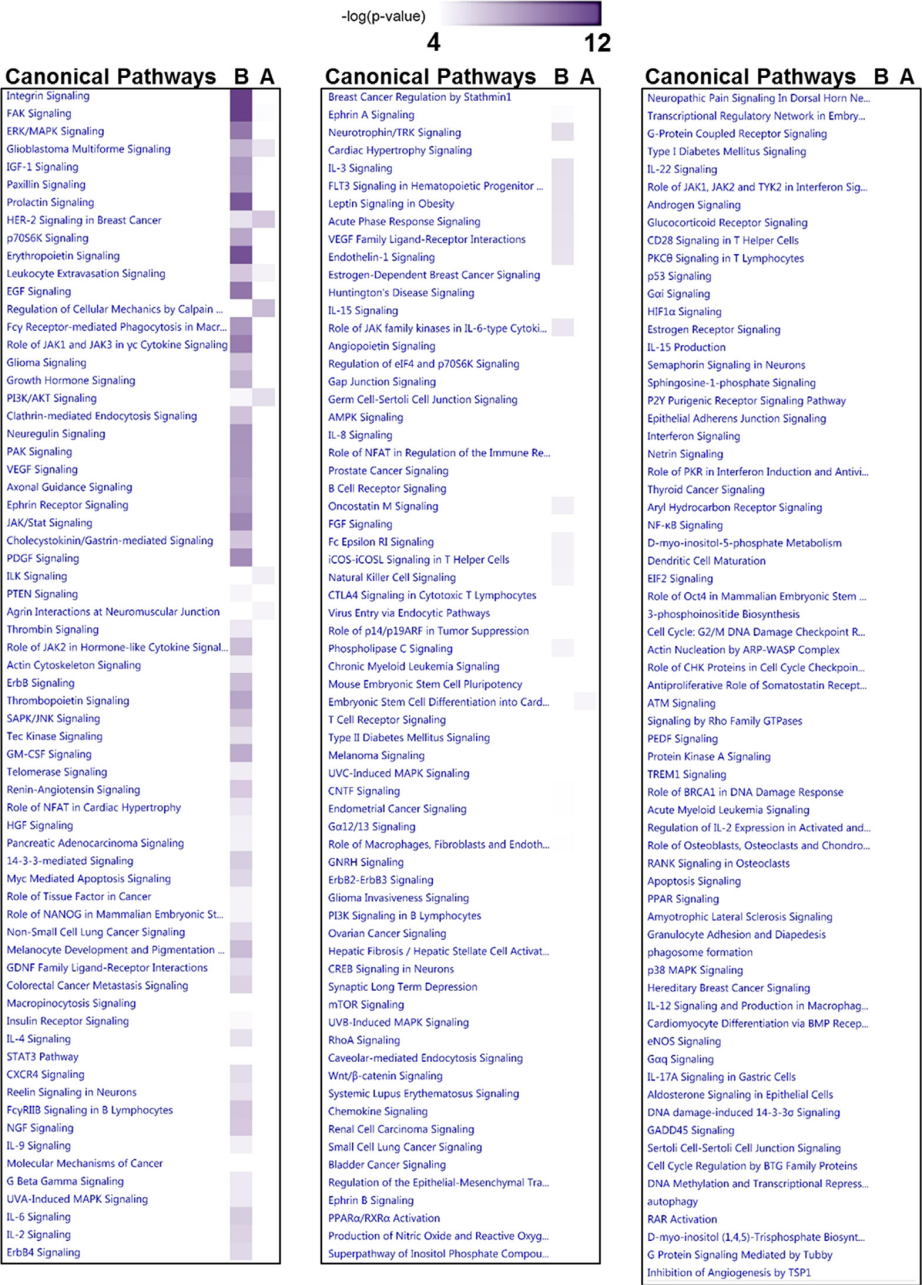
Enrichment of canonical signaling pathways by IDR-interacting proteins of FAK1 interactome. Mapped interactome of FAK1 was divided into two interactomes. A – Ordered region-interacting proteins; B – IDR-interacting proteins. Differentially enriched canonical pathways were identified by using comparison analysis feature of IPA. Raw data is described in Supplementary Table 9.

Experimentally validated protein-protein interaction (PPI) data of the 36 kinases and that of FAK1 interacting proteins was assembled using manual data curation and various softwares including as described previously [4] (Supplementary Tables 5–8). PPI network was constructed and visualized using Cytoscape [16] (Fig. 4). Network analysis was performed to identify topologically significant hubs from the PPI networks using Network Analyzer [17] and CentiScaPe plug in tools [18] (Fig. 5). Further, canonical signaling pathways by IDR-interacting proteins of FAK1 interactome were enriched (Fig. 6).

## Appendix A Supplementary material

.

**Supplementary Table 1: 25% of the total amino acid residues of the kinome are intrinsically disordered:** Analysis of the disorder prediction of human kinome identified that 25% of the total amino acids of the human kinome are intrinsically disordered. Furthermore, different groups of kinases have different percent disorder. **Supplementary Table 2: IDRs evolve at a faster rate in the human kinome:** Relative evolution rates were calculated for the hybrid kinases, their KDs, and their IDRs. IDRs have the highest rates ratio indicating faster rate of evolution. 211 kinases had at least two IDRs. Analysis of evolution of multiple IDRs within a single kinase revealed that IDR near KD evolves at a slower rate than IDR further away from the KD. **Supplementary Table 3: Evolutionary Rates of IDRs in the human kinome:** Relative evolution rates for each of the IDRs in a given kinase are listed. **Supplementary Table 4: 65% of kinases have IDRs flanking their kinase domains:** We identified 328 kinases (65% of the human kinome) that had an IDR within 100 amino acids of their kinase domains.

**Supplementary Table 5: 36 Kinases have IDRs extending into their kinase domains:** Further analysis of the 328 kinases with IDRs flanking their KDs revealed 36 kinases with IDRs either extending into or spanning their KDs. **Supplementary Table 6: 36 Kinases participate in 959 interactions:** Experimentally validated PPIs of 36 kinases are derived to reveal 959 interactions. Column A depicts Uniprot IDs of 36 kinases and Column C depicts Uniprot IDs of proteins interacting with each of the 36 kinases. **Supplementary Table 7: 78% of proteins in the primary interactome of FAK1 are hybrid proteins:** Experimentally validated FAK1 interacting proteins are analyzed to predict intrinsic disorder using Pondr-FIT. We identify 77 of 98 proteins (78%) as hybrid proteins with at least one IDR of 25 amino acids or longer. % DO (disorder) is calculated as a percentage of disordered residues in a given protein. **Supplementary Table 8: 76% of proteins in the secondary interactome of FAK1 are hybrid proteins:** Experimentally validated interactions of the eleven cytoskeletal hybrid proteins are derived and analyzed to reveal 1784 interactions by 1154 proteins. These 1154 proteins are analyzed to predict their percent disorder revealing 882 proteins (76%) as hybrid proteins. We also identify 352 proteins that interact with at least two of the eleven cytoskeletal hybrid proteins. **Supplementary Table 9: Comparison of molecular functions enriched by Order regions-interacting (A) or IDR-interacting (B) Proteins:** Molecular functions enriched by either Interactome A (interactors using Ordered Regions) or Interactome B (interactors using IDRs) were compared against each other. Lower p-values reflect increased enrichment of a given molecular function compared between the two interactomes. Supplementary material

## Footnotes

Appendix A Supplementary data associated with this article can be found in the online version at doi:10.1016/j.dib.2016.11.099.
