# Supplementary material for: Data on evolution of intrinsically disordered regions of the human kinome and contribution of FAK1 IDRs to cytoskeletal remodeling
Source: Data Brief. 2016 Dec 8;10:315–24. doi: 10.1016/j.dib.2016.11.099 (PMC5157709; doi:10.1016/j.dib.2016.11.099)
Supplement: Supplementary file 1 — Supplementary material [file mmc1.docx]

Conflicts of interest: None
